# Perioperative changes in hemoglobin levels during major hepatopancreatic surgery in transfused and non-transfused patients

**DOI:** 10.1177/1457496920964362

**Published:** 2020-10-29

**Authors:** J.P. Lammi, Matti Eskelinen, Jarno Tuimala, Tuomas Selander, Juha Saarnio, Tuomo Rantanen

**Affiliations:** Department of Surgery, Kuopio University Hospital, Kuopio, Finland; Department of Surgery, Kuopio University Hospital, Kuopio, Finland; School of Medicine, University of Eastern Finland, Kuopio, Finland; Not affiliated, Helsinki, Finland; School of Medicine, University of Eastern Finland, Kuopio, Finland; Department of Surgery, Oulu University Hospital, Oulu, Finland; School of Medicine, University of Eastern Finland, P.O. Box 100, FI-70029 KYS, Finland

**Keywords:** Major hepatic surgery, major pancreatic surgery, Hb level, perioperative Hb levels, blood transfusion

## Abstract

**Background:**

Several studies have shown that restrictive transfusion policies are safe. However, in clinical practice, transfusion policies seem to be inappropriate. In order to assist in decision-making concerning red blood cell transfusions, we determined perioperative hemoglobin (Hb) levels during major pancreatic and hepatic operations.

**Methods:**

Patients who underwent major pancreatic or hepatic resections between 2002 and 2011 were classified into the transfused (TF+) and non-transfused (TF) groups. The perioperative Hb values of these patients were evaluated at six points in time.

**Results:**

The study included 1596 patients, of which 785 underwent pancreatoduodenectomy, 79 total pancreatectomy, and 732 partial hepatectomy. Similar perioperative changes in Hb levels were seen in all patients regardless of whether they received a blood transfusion. In patients undergoing pancreatoduodenectomy and total pancreatectomy, *the median of the lowest measured hemoglobin values was 89.2 g/L and in partial hepatectomy patients 92.6 g/L, and these were assumed to be the trigger points for red blood cell transfusion.*

**Conclusions:**

Despite guidelines on blood transfusion thresholds, restrictive blood transfusion policies were not observed during our study period. After major pancreatic and hepatic surgery, Hb levels recovered without transfusions. This should encourage clinicians to obey the restrictive blood transfusion policies after major hepatopancreatic surgery.

## Introduction

Red blood cell transfusion (RBCT) is one of the most frequently used therapies worldwide and is associated with benefits, risks, and costs. Hepatopancreatobiliary (HPB) surgery carries a significant risk of hemorrhage and RBCT. In a cohort of 26,827 patients undergoing HPB surgery, 25.7% received RBCT^1^, while in a recent meta-analysis on post-pancreatectomy hemorrhage, the incidence of hemorrhages ranged from 3% to 16% ^2^. Previously published studies have reported blood loss ranging from 210 to 700 mL after open liver resection and from 127 to 450 mL after laparoscopic liver resection^3–5^. Following hepatic surgery, 25.2%–56.8% of patients receive RBCT ^6^. There are conflicting data about the effects of RBCT on oncological results; some studies show a poorer outcome after RBCT^7–9^, while some demonstrate no association between RBCT and overall survival^10^. Nevertheless, whether blood transfusions have an impact on oncologic outcomes or not, transfusions are costly and risky, with complications including allergic reactions, transfusion-associated circulatory overload (TACO), hemolytic and febrile transfusion reactions, and thrombotic complications^11^, and every effort to decrease unnecessary transfusions should be made.

For decades, clinicians applied the 100/30 rule as a transfusion trigger to keep patients’ Hb levels above 100 g/L and the hematocrit level above 30%. In the 1980s, public fear of HIV infection and efforts to decrease expenses provoked a re-examination of transfusion practices. The 1988 National Institutes of Health Consensus Conference on perioperative blood cell transfusions suggested that no single criterion should be used as an indication for red cell component therapy^12^.

The Transfusion Requirement in Critical Care (TRICC) trial is the most cited clinical trial evaluating RBCT thresholds. There were no significant differences between the liberal (threshold <100 g/L) and restrictive (threshold <70 g/L) groups in short- and long-term mortality, infections, or days on a ventilator^13^.

According to the AABB guidelines, a restrictive RBCT threshold of 70 g/L is recommended for hospitalized adult patients who are hemodynamically stable, including critically ill patients, rather than a threshold of 100 g/L. A restrictive RBCT threshold of 80 g/L is recommended for patients undergoing orthopedic or cardiac surgery and those with pre-existing cardiovascular disease^14^.

In 2018, the Frankfurt Consensus Conference presented recommendations concerning patient blood transfusion practices. As regards RBCT thresholds, two strong clinical recommendations were formulated. First, the panel recommended a restrictive transfusion threshold (Hb concentration <70 g/L) for critically ill but clinically stable intensive care patients. The second recommendation was for patients undergoing cardiac surgery, for whom the panel recommended a threshold of 75 g/L. However, there were no good-quality data on most patient groups, including patients undergoing HPB surgery, to issue further recommendations on transfusion thresholds^15^.

Various studies have demonstrated vast variation in hospitals’ perioperative transfusion practices. The Sanguis Study Group examined the use of blood products in 43 European hospitals. The percentage of surgical patients who were transfused with red blood cell units ranged from 0% to 79% in right and left hemicolectomies, from 17% to 100% in coronary artery bypass graft implantations and from 29% to 100% in total hip replacement procedures^16^. Nineteen years later, similar results were demonstrated in total hip replacement, colectomy, and pancreatoduodenectomy (PD) procedures^17^.

We examined the perioperative Hb level behavior during major hepatic and pancreatic resections in patients who received a blood transfusion (TF+) and in those who did not (TF−). Our goal was to elucidate the perioperative Hb level changes in TF+ and TF− patients and to encourage clinicians to observe the restrictive transfusion threshold guidelines^14^. To the best of our knowledge, this is the first study on perioperative Hb levels including both TF+ and TF− patients in HPB surgery.

## Materials and Methods

### Data

The Finnish Red Cross Blood Service (FRCBS) is the blood service provider for all Finnish hospital districts. A joint national registry (VOK) was established by the FRCBS in 2002 to serve as a benchmarking effort to evaluate and develop blood transfusion practices in Finland. The registry was updated on a regular basis between 2002 and 2011 by, at most, five central and five university hospitals. The registry was permanently dissolved in 2011, and adding new data to the registry is no longer possible.

The data collection system has been described in more detail earlier^18^. In short, patients undergoing predefined surgical procedures or having certain diagnosis codes were collected into the registry. The database contains data on patient characteristics, diagnoses, surgical procedures, laboratory results, and blood transfusions.

For technical reasons, including data from all 10 hospital districts collaborating in the VOK registry was not possible. Therefore, for this study, data were acquired for eight hospital districts involved in the VOK registry. Patients who underwent PD, total pancreatectomy (TP), or partial hepatectomy (PH) between 2002 and 2011 were extracted. These operations were selected due to their high incidence of perioperative RBCTs^1,6^. PD and TP were analyzed simultaneously due to the similarity of the procedures.

The data were divided into TF+ and TF− groups on the basis of RBCT status. In addition to the RBCT status, the patient’s age at the time of the operation, as well as sex, the American Society of Anesthesiologists (ASA) class, operational status, the length and number of the operations, and the length of the hospital stay were used for further analyses. *We also carried out a subgroup analysis of TF+ patients: the patients were divided into two groups based on whether they had an Hb level below 80 g/L at some point of the inpatient episode. The cut-off of 80 g/L was selected because of the small number of patients (59 patients) whose Hb level was below 70 g/L, which is the suggested threshold in the guidelines.*

To determine the perioperative Hb levels, we searched the VOK registry, as regards the eight hospitals included in this study, for the patients’ last recorded Hb value before the inpatient episode, measured no more than 6 months before the episode, as well as the first Hb value of the inpatient episode, the Hb value immediately before the operation, the Hb value on the morning of the first postoperative day, the lowest Hb value during the hospital stay, and the last Hb value before the patients’ discharge. Hb drift is defined as an upward or downward trend in Hb concentration perioperatively.

### Statistical Analyses

Measurements were compared between TF+ and TF− patients using the Mann–Whitney *U* test, or a test for proportions, where applicable.

The Hb drift was modeled with a linear mixed model with Gaussian errors. Since Hb measurements are unimodal and roughly symmetric around the mean, the Gaussian error is an acceptable choice for the model. For trend tests using the mixed modeling framework, the phase of the episode (Hb determinations before the episode, first of the episode, prior to the operation, postoperative morning, lowest postoperatively, and the last of the episode) was included as the predictor of Hb levels. The patient-specific effect was modeled as a random intercept term. These results are presented in the legends of the figures. Only cases with Hb measurements available for all the mentioned phases were included in the trend analyses.

All statistical analyses were carried out using the R software version 3.5.2 (R Foundation for Statistical Computing, Vienna, Austria). The linear mixed models were fitted using the nlme add-on package in R (R package version 3.1-137). A level of two-tailed p < 0.05 was considered statistically significant.

### Ethics

The right to use the VOK registry in this study was acquired from the individual hospital districts that own the data, and from the national authority for health-related registry research (National Institute for Health and Welfare). The study was approved by the ethical committee of Kuopio University Hospital.

## Results

### Patient Characteristics

Overall, 1596 patients were included. Liver resections included 131 open non-anatomical excisions, seven laparoscopic excisions, 45 open wedge resections, 117 excisions of a single segment, 207 excisions of two segments, 64 left hemihepatectomies, 109 right hemihepatectomies, 11 extended right hemihepatectomies, and 45 other excisions of three or more segments. Pancreas resections included 24 duodenum-preserving TP, 785 PD, and 55 TP. The baseline demographic characteristics and surgical data of the study patients are shown in Tables 1 and 2.

**Table 1.** Baseline demographics and surgical data for the study groups of patients who underwent pancreaticoduodenectomy or total pancreatectomy. Values are presented as mean values (standard deviation) or the number of cases. Results are shown for all patients, for those with a blood transfusion (TF+) and for those without blood transfusions (TF−). The distributions of ASA classes I–IV are presented as percentages of the number of patients within TF+ and TF−. Percentages are calculated for all patients of a given variable.

Variable	All (N = 864)	TF+ (N = 564)	TF− (N = 300)	p-value
Age, years	63.4 (11.5)	64.4 (11.1)	61.5 (12.2)	<0.001
Sex (male/female)	460/404	287/277	173/127	0.067
ASA				
I	50	22 (44.0)	28 (56.0)	0.003
II	295	170 (57.6)	125 (42.4)	0.002
III	366	259 (70.8)	107 (29.2)	<0.001
IV	29	27 (93.1)	2 (6.9)	0.002
V	0	0	0	NA
ASA not available	124	86 (69.4)	38 (30.6)	0.353
Elective surgery	804	515 (64.1)	289 (35.9)	0.009
Emergency surgery	60	49 (81.7)	11 (18.3)	0.009
Operative time, min	305 (79)	319 (83)	278 (65)	<0.001
Operations per patient during episode*	1.13 (0.49)	1.18 (0.58)	1.03 (0.20)	<0.001
Length of hospital stay, days	15.9 (14.6)	18.2 (17.0)	11.8 (6.5)	<0.001

ASA: American Society of Anesthesiology Physical Status Classification System; NA: not available.

* Any operations during inpatient episode; the median of the number of operations is always 1.

**Table 2.** Baseline demographics and surgical data for the study groups of patients who underwent partial hepatectomy. Values are presented as mean values (standard deviation) or the number of cases. Results are shown for all patients, for those with a blood transfusion (TF+) and for those without a blood transfusion (TF−). Percentages are calculated for all patients of a given variable. For a small number of patients, partial hepatectomies were performed repeatedly, and the inpatient episode–specific variables, such as ASA classification, are reported per episode in the table. Hence, the numbers do not necessarily add up to the overall number of patients. Because the amount of repeated operations was so small, they were not excluded from the data set.

Variable	All (N = 732)	TF+ (N = 359)	TF− (N = 373)	p-value
Age, years	59.5 (15.6)	58.7 (18.0)	60.3 (12.8)	0.785
Sex (male/female)	378/354	169/190	209/164	0.012
ASA				
I	29	5 (17.2)	24 (82.8)	0.001
II	252	110 (43.7)	142 (56.3)	0.054
III	386	202 (52.3)	184 (47.7)	0.046
IV	41	25 (61.0)	16 (39.0)	0.146
V	10	8 (80.0)	2 (20.0)	0.094
ASA not available	14	9 (64.3)	5 (35.7)	0.378
Elective surgery	707	343 (48.5)	364 (51.5)	0.123
Emergency surgery	24	16 (66.7)	8 (33.3)	0.123
Operative time, min	231 (119)	272 (128)	190 (93)	<0.001
Operations per patient during episode*	1.03 (0.25)	1.06 (0.36)	1.00 (0.00)	<0.001
Length of hospital stay, days	6.8 (6.2)	8.4 (7.8)	5.4 (3.8)	<0.001

ASA: American Society of Anesthesiology Physical Status Classification System.

* Any operations during inpatient episode; the median of the number of operations is always 1.

Of the 864 PD and TP patients, 564 (65.3%) received RBCTs. The TF+ patients were slightly older (64.4 vs 61.5 years, p < 0.001), and their ASA classification was higher than that of the TF− patients. The operative times and inpatient episodes were also longer (319 vs 278 min, p < 0.000; 18.2 vs 11.8 days, p < 0.001) among the TF+ patients. The TF+ patients also underwent a higher number of reoperations during the inpatient episodes. The PD and TP patients in the TF+ group received a mean 5.4 (standard deviation (SD) 7.8.) RBCTs (median 3 and interquartile range (IQR) 2–6).

Of the 732 PH patients, 359 (49.0%) patients received an RBCT. The TF+ patients’ ASA classification was higher than that of the TF− patients. The operative time (272 vs 190 min, p < 0.001) and the length of the inpatient episode (8.4 vs 5.4 days, p < 0.001) were longer in the TF+ group. The TF+ patients underwent a higher number of reoperations during the inpatient episodes. The mean 4.3 (SD 4.6.) RBCTs were given to TF+ PH patients (median 3 and IQR 2–5).

### Perioperative Changes in Hb Levels

The mean perioperative Hb values are shown in Tables 3 and 4. Hb values demonstrated a similar pattern in all groups, but all pre- and postoperative Hb values were lower among the TF+ patients than the TF– patients. A downward drift before the inpatient episode was followed by an upward drift before the operation. After the operation, there was a downward drift in all groups, which was followed by an upward drift before discharge.

**Table 3.** Perioperative hemoglobin values in patients undergoing pancreaticoduodenectomy and total pancreatectomy. Values are presented as mean values (standard deviation). Results are shown for all patients, for those with a blood transfusion (TF+) and those without a blood transfusion (TF–).

Hemoglobin measurement	All (N = 864)	TF+ (N = 564)	TF− (N = 300)	p-value
Before inpatient episode	129.8 (15.2)	127.1 (15.5)	135.3 (13.1)	<0.001
First of inpatient episode	119.3 (18.6)	117.1 (17.9)	123.8 (19.1)	<0.001
Before operation	125.7 (15.9)	122.0 (15.2)	133.9 (14.1)	<0.001
First postoperative morning	108.4 (13.5)	107.2 (14.1)	110.8 (12.0)	<0.001
Lowest of the episode	92.2 (13.9)	89.2 (14.4)	98.2 (10.5)	<0.001
Last of the episode	110.4 (12.3)	110 (12.2)	110.3 (12.9)	0.936

**Table 4.** Perioperative hemoglobin values in partial hepatectomy patients. Values are presented as mean values (standard deviation). Results are shown for all patients, for those with a blood transfusion (TF+) and those without a blood transfusion (TF−).

Hemoglobin measurement	All (N = 732)	TF+ (N = 359)	TF− (N = 373)	p-value
Before inpatient episode	130.3 (16.0)	124.5 (17.4)	135.4 (12.7)	<0.001
First of inpatient episode	125.5 (17.9)	119.7 (18.7)	131.1 (15.3)	<0.001
Before operation	129.1 (17.1)	123.5 (18.4)	134.4 (13.7)	<0.001
First postoperative morning	109.7 (13.8)	106.4 (13.9)	113.2 (12.7)	<0.001
Lowest of the episode	99.0 (14.6)	92.6 (12.7)	105.5 (13.5)	<0.001
Last of the episode	113.4 (15.6)	108.3 (13.5)	118.3 (16.0)	<0.001

PD and TP patients in the TF− group experienced the greatest Hb drop (35.7 g/L) after the operation. In the TF+ group, PD and TP patients had a total Hb decline of 32.8 g/L. The Hb levels of TF− PH patients declined by 28.9 g/L and those of TF+ PH patients by 30.9 g/L postoperatively. TF+ PD and TP patients had the lowest Hb levels (89.2 g/L) after the operation and also the greatest drop (18 g/L) after the measurement on the first postoperative morning.

Before discharge, all groups experienced an upward drift. A recovery of the Hb levels was seen in all groups with or without blood transfusions. The TF+ PD and TP group experienced the greatest rise of 20.8 g/L, while TF− PD and TP patients had a 12.1 g/L increase. The TF+ PH patients experienced a 15.7 g/L increase and the TF− PH patients a 12.8 g/L increase in their Hb levels.

We also carried out a subgroup analysis of TF+ patients. The patients were divided into two groups based on whether their Hb level measured below 80 at some point of the inpatient episode. The Hb values of these patients are shown in Tables 5 and 6. Of the 564 TF+ PD and TP patients, 117 (20.7%) had an Hb level below 80 g/L at some point of the inpatient episode. Only 34 (0.9%) of the 359 TF+ PH patients had an Hb level under 80 g/L at some point of the inpatient episode. However, all of Hb values for these patients were not available.

**Table 5.** Perioperative hemoglobin values in all TF+ patients undergoing pancreaticoduodenectomy and total pancreatectomy and in those in whom the lowest hemoglobin of the inpatient episode was either ⩽80 g/L or >80 g/L. Values are presented as mean values (standard deviation). The number of patients differs between total and subgroups because of missing data.

Hemoglobin measurement	TF+ (N = 564)	Hb (⩽80) (N = 117)	Hb (>80) (N = 410)	p-value
Before inpatient episode	127.1 (15.5)	128.0 (16.1)	127.1 (15.3)	0.606
First of inpatient episode	117.1 (17.9)	111.5 (20.4)	118.6 (16.9)	<0.001
Before operation	122.0 (15.2)	124.4 (14.8)	121.6 (15.3)	0.120
First postoperative morning	107.2 (14.1)	102.0 (15.6)	108.8 (13.2)	<0.001
Lowest of the episode	89.2 (14.4)	69.7 (10.7)	94.8 (9.8)	<0.001
Last of the episode	110 (12.2)	106.3 (14.3)	111.7 (11.0)	<0.001

**Table 6.** Perioperative hemoglobin values in all TF+ patients undergoing partial hepatectomy and in those in whom the lowest hemoglobin of the inpatient episode was either ⩽80 g/L or >80 g/L. Values are presented as mean values (standard deviation). The number of patients differs between total and subgroups because of missing data.

Hemoglobin measurement	TF+ (N = 359)	Hb (⩽80) (N = 34)	Hb (>80) (N = 208)	p-value
Before inpatient episode	124.5 (17.4)	113.7 (22.9)	126.1 (16.0)	0.009
First of inpatient episode	119.7 (18.7)	107.2 (22.1)	120.2 (17.4)	0.002
Before operation	123.5 (18.4)	110.8 (22.5)	124.7 (16.9)	0.002
First postoperative morning	106.4 (13.9)	95.9 (13.2)	108.1 (13.3)	<0.001
Lowest of the episode	92.6 (12.7)	73.8 (7.1)	95.7 (10.6)	<0.001
Last of the episode	108.3 (13.5)	99.6 (14.8)	108.2 (10.0)	0.002

## Discussion

Our study has two main findings. First, all patient groups, regardless of whether they received a blood transfusion, displayed similar changes in Hb levels and an almost identical postoperative upward drift. The Hb values recovered after the operation to almost the same level in both TF− and TF+ patients. Second, the transfusion policies were very liberal in the hospitals included in the study. To the best of our knowledge, this study, albeit it includes only HPB patients, is the first study on perioperative Hb levels including both TF+ and TF− patients.

Perioperative Hb levels have been investigated previously in two studies. George et al. showed in cardiac surgery an average postoperative Hb drop of 11 ± 14 g/L. 80% of the patients recovered from their Hb nadir to some degree. Cardiac surgery, however, differs from the procedures we examined. For example, in cardiac surgery, a cardiopulmonary bypass was a strong predictor of an Hb decline. In addition, some patients received autologous transfusions, which can clearly have an impact on the Hb drift^19^.

Grant et al. examined postoperative Hb levels in 11 common surgical procedures. The data were further analyzed for two procedures with a large Hb drift, PD (n = 82) and lumbar spinal fusion (n = 74). The authors found that the primary predictor of greater Hb drift was a surgical procedure with greater intraoperative intravenous (IV) fluid and blood requirements. The mean Hb decline was 20–25 g/L, which was followed by a 6 g/L recovery in two-thirds of the patients ^20^.

Our results are similar to these two studies, which, however, have some drawbacks—one of which was that patients who received a postoperative TF were excluded. In our study, similar changes in Hb levels were found among both TF− and TF+ patients in all procedure groups.

The reasons behind the changes in Hb levels remain unknown, since our study was not designed to address this aspect. George et al.^19^ speculated on the reasons for the Hb drift in their study; the possible causes could include impaired erythropoiesis and a decreased survival of transfused red blood cells. In our opinion, the best explanation for Hb drift could be postoperative hemodilution due to liberal fluid replacement with intraoperative crystalloids. As Grant et al. showed, patients who have undergone PD are approximately 8 L positive for fluid balance^20^. Third-space losses refer to the abnormal accumulation of fluids into extracellular and extravascular spaces. During surgery, only approximately 25%–30% of infused crystalloids remain in the circulation, while the remaining 70%–75% shift to the interstitium. In our opinion, this could explain the entire pattern of Hb drift. This was also pointed out by George et al., as cardiopulmonary bypass causes an inflammation, leading to third-space losses of fluids^19^.

We found a downward Hb drift before the inpatient episode, which may be explained by cancer-related anemia^21^. We also found an upward drift immediately before the operation. Traditionally, patients undergo fast and fluid intake restrictions before an operation. One can only speculate whether this preoperative fasting, leading to hemoconcentration, could have had an impact on the preoperative increase in Hb levels.

Another finding was that restrictive transfusion policies were not followed, as our results show a practice of perioperative over-transfusion. In PD and TP patients, the median assumed transfusion trigger was an Hb level of 89.2 g/L and in PH patients 92.6 g/L. The lowest Hb value of the inpatient episode was assumed to be the transfusion trigger for each patient. The subgroup analysis of TF+ patients reveals similar results. In the TF+ group, 20.7% of the PD and TP patients and only 0.9% of the PH patients had an Hb level of under 80 g/L at some point of the inpatient episode. However, all of the Hb values for these patients were not available to us. The lowest Hb values in these subgroups were 69.7 g/L (PD and TP patients) and 73.8 g/L (PH patients) (Tables 5 and 6). It is likely that most of the patients in these subgroups would have also received an RBCT according to a restrictive transfusion *policy*.

The patients left the hospital with a median Hb of approximately 110 g/L. The reasons for over-transfusing are most likely human. During the operation, *anesthesiologists may* use pre-emptive RBCTs for anticipated bleeding. *After the operation, surgeons want their patients to feel better and think that RBCTs could help. Clinicians also need to consider patient-specific factors and may not feel the need to adhere strictly to restrictive transfusion policies.*

Many institutions are currently successful in reducing the overall blood utilization by applying a restrictive (70 g/L) Hb trigger policy. We have previously shown that the lowering of the transfusion trigger point and optimal use of RBCTs occurred at high-volume centers^22^. The centralization of complex surgery might lead to a better use of RBCTs.

The main limitation of this study is that the registry-based nature does not allow us to analyze all possible interesting aspects. For example, the assessment of the patient’s condition before the operation is rather limited, mainly consisting of the ASA classification only. In addition, any possible neoadjuvant chemotherapy and the use of any medication or preoperative iron deficiency affecting the Hb level could not be ascertained. We also did not have data on intraoperative bleeding during the operations. *The lowest measured Hb value was assumed to be the transfusion threshold. This assumption is based on the fact that clinicians usually start giving RBCTs when a certain Hb value trigger point is reached. This assumption does not apply in the case of major bleeding when RBCTs are given without determining Hb values. Furthermore, it is possible that, in some cases, the Hb values have been declining after the beginning of the RBCTs, as data on the stages of the episode at which the RBCTs were given were not available to us.*

Despite the guidelines on blood transfusion thresholds, restrictive blood transfusion policies were not observed during our study period. As reinforced by the 2018 Consensus Conference, a restrictive transfusion policy is suitable for most patients undergoing major elective surgery. We show herein that, after major pancreatic and hepatic surgery, Hb levels recover without transfusions. This should encourage clinicians to obey the restrictive blood transfusion policies after major hepatopancreatic surgery.

## References

[1] Lucas DJ Schexneider KI Weiss M , et al. Trends and risk factors for transfusion in hepatopancreatobiliary surgery. J Gastrointest Surg 2014;18;719–728. 24323432 10.1007/s11605-013-2417-9

[2] Floortje van Oosten A Smits FJ van den Heuvel DAF , et al. Diagnosis and management of postpancreatectomy hemorrhage: A systematic review and meta-analysis. HPB 2019;21(8):953–961. 30962134 10.1016/j.hpb.2019.02.011

[3] Cipriani F Rawashdeh M Stanton L , et al. Propensity score-based analysis of outcomes of laparoscopic versus open liver resection for colorectal metastases. Br J Surg 2016;103:1504–1512. 27484847 10.1002/bjs.10211

[bibr4-1457496920964362] Lewin JWO O’Rourke NA Chiow AKH , et al. Long-term survival in laparoscopic vs open resection for colorectal liver metastases: Inverse probability of treatment weighting using propensity scores. HPB 2016;18(2):183–191. 26902138 10.1016/j.hpb.2015.08.001 PMC4814613

[5] Hasegawa Y Nitta H Sasaki A , et al. Long-term outcomes of laparoscopic versus open liver resection for liver metastases from colorectal cancer: A comparative analysis of 168 consecutive cases at a single center. Surgery 2015;157(6):1065–1072. 25791030 10.1016/j.surg.2015.01.017

[6] Latchana N Hirpara DH Hallet J , et al. Red blood cell transfusion in liver resection. Langenbecks Arch Surg 2019;404:1–9. 10.1007/s00423-018-1746-2 30607533

[7] Makino Y Yamanoi A Kimoto T , et al. The influence of perioperative blood transfusion on intrahepatic recurrence after curative resection of hepatocellular carcinoma. Am J Gastroenterol 2000;95(5):1294–1300. 10811342 10.1111/j.1572-0241.2000.02028.x

[bibr8-1457496920964362] Vamvakas EC. Perioperative blood transfusion and cancer recurrence: Meta-analysis for explanation. Transfusion 1995;35(9):760–768. 7570938 10.1046/j.1537-2995.1995.35996029162.x

[9] Koch M Antolovic D Reissfelder C , et al. Leucocyte-depleted blood transfusion is an independent predictor of surgical morbidity in patients undergoing elective colon cancer surgery-a single-center analysis of 531 patients. Ann Surg Oncol 2011;18(5):1404–1411. 21153884 10.1245/s10434-010-1453-x

[10] Warschkow R Güller U Köberle D , et al. Perioperative blood transfusions do not impact overall and disease-free survival after curative rectal cancer resection: A propensity score analysis. Ann Surg 2014;259:131–138. 23470578 10.1097/SLA.0b013e318287ab4d

[11] Shot report, 2018, www.shotuk.org

[12] Consensus conference. Perioperative red blood cell transfusion. JAMA 1988;260:2700–2703. 3054179

[13] Hébert PC Wells G Blajchman MA , et al. A multicentre randomized, controlled clinical trial of transfusion requirements in critical care. Transfusion requirements in critical care -investigators, Canadian critical care Trials Group. N Engl J Med 1990;340:409–417. 10.1056/NEJM199902113400601 9971864

[14] Carson JL Guyatt G Heddle NM , et al. Clinical practice guidelines from the AABB: Red blood cell transfusion thresholds and storage. JAMA 2016;316:2025–2035. 27732721 10.1001/jama.2016.9185

[15] Mueller MM Van Remoortel H Meybohm P , et al. Patient blood management: Recommendations from the 2018 Frankfurt Consensus Conference. JAMA 2019;321:983–997. 30860564 10.1001/jama.2019.0554

[16] The Sanguis Study Group. Use of blood products for elective surgery in 43 and European hospitals. Transfus Med 1994;4:251–268. 7889138

[17] Qian F Osler TM Eaton MP , et al. Variation of blood transfusion in patients undergoing major noncardiac surgery. Ann Surg 2013;257(2):266–278. 22801086 10.1097/SLA.0b013e31825ffc37

[18] Palo R Ali-Melkkila T Hanhela R , et al. Development of permanent national register of blood component use utilizing electronic hospital information systems. Vox Sang 2006;91(2):140–147. 16907875 10.1111/j.1423-0410.2006.00814.x

[19] George TJ Beaty CA Kilic A , et al. Hemoglobin drift after cardiac surgery. Ann Thorac Surg 2012;94(3):703–709. 22609121 10.1016/j.athoracsur.2012.03.038 PMC3610599

[20] Grant MC Whitman GJ Savage WJ , et al. Clinical predictors of postoperative hemoglobin drift. Transfusion 2014;54(6):1460–1468. 24236570 10.1111/trf.12491

[21] Gilreath JA Stenehjem DD Rodgers GM. Diagnosis and treatment of cancer-related anemia. Am J Hematol 2014;89(2):203–212. 24532336 10.1002/ajh.23628

[22] Lammi JP Eskelinen M Tuimala J , et al. Blood transfusions in major pancreatic surgery: A 10-year cohort study including 1404 patients undergoing pancreatic resections in Finland. Scand J Surg 2019;108(3):210–215. 30458689 10.1177/1457496918812207

